# Epidemiological analysis of axillary apocrine bromhidrosis in China: a survey from Chinese higher education students

**DOI:** 10.3389/fmed.2023.1232744

**Published:** 2023-11-06

**Authors:** Lei Zhang, Jiaqi Cheng, Cangyu Wang, Junhong Zhao, Cuiping Zhang, Haihong Li

**Affiliations:** ^1^Department of Mental Health Center, The Seventh Affiliated Hospital, Sun Yat-sen University, Shenzhen, Guangdong Province, China; ^2^Laboratory of Wound Repair and Dermatologic Surgery, Taihe Hospital, Hubei University of Medicine, Shiyan, Hubei Province, China; ^3^Research Center for Tissue Repair and Regeneration Affiliated to the Medical Innovation Research Department and Fourth Medical Center of PLA General Hospital, Beijing, China; ^4^Department of Burns and Plastic Surgery, The Seventh Affiliated Hospital, Sun Yat-sen University, Shenzhen, Guangdong Province, China

**Keywords:** axillary apocrine bromhidrosis (AAB), epidemiological survey, Chinese population, prevalence, SCL-90, higher education students

## Abstract

**Background:**

There are few epidemiological data on axillary apocrine bromhidrosis (AAB) in the Chinese population, making it impossible to accurately estimate its prevalence or impact on individuals.

**Objective:**

To estimate the prevalence of AAB in China, and to survey and compare the psychological status of individuals with and without AAB.

**Methods:**

Students in several universities in China were surveyed online for AAB, and the prevalence of AAB was calculated. The Symptom Checklist 90 (SCL-90) was used to evaluate the psychological status.

**Results:**

The prevalence of AAB in the surveyed students was 7.5% (194/2571). The projected number of Chinese higher education students with AAB was about 3 million. The onset age of AAB was mainly between 11 and 20 years old (79.90%, 155/194). 68.04% (132/194) of individuals with AAB had a positive family history, and 60.30% (117/194) had wet earwax. Individuals with AAB often felt depression, anxiety, loneliness and social alienation, and scored significantly higher on the nine primary psychological symptom dimensions than individuals without AAB.

**Conclusion:**

AAB affects a small proportion but large numbers of Chinese population. China and the West or East-Asia and the West have different perception, recognition and treatment preferences for AAB.

## 1. Background

Body odor is present in all animals (1). Although it played an important role in early humans, it is generally considered to be an unpleasant odor among many human cultures (2, 3). Many parts of the human body, such as the mouth, axillary and foot, produce odors, but axillary odor may be the most powerful and impressive (4). Axillary apocrine bromhidrosis (AAB) is a clinical diagnosis characterized by an unpleasant pungent odor from the axillary apocrine sweat glands (2). Whether an individual suffers from AAB depends primarily on the patient’s self-assessment and/or the examiner’s judgment (2). Although researchers have recommended some objective indicators for diagnosing AAB, such as body odor potential, body odor evoked scale, odor sensors, and pH, these indicators have not yet been used as diagnostic criteria for AAB (5–7). Odor is a subjective factor, therefore, the diagnosis of AAB remains a subjective diagnosis. Apocrine sweat glands do not become active until puberty, so axillary odor is more common in individuals after puberty (2). AAB has a strong genetic basis, which is very common in Western countries and is therefore not considered a normal phenomenon (8, 9). However, in Asian countries, especially East Asian countries, such as China, Japan and South Korea, the prevalence of AAB is not high, and it is generally considered a disease (10). Although AAB does not cause major health problems, it can lead to serious social and psychological problems (2, 10).

The Symptom Checklist-90 (SCL-90) is a brief self-report psychometric instrument to evaluate a broad range of psychological problems and psychopathological symptoms (11, 12). The scale consists of nine dimensions with 90 items in total. The nine symptom dimensions evaluated are as follows: somatization, obsessive compulsive symptoms, interpersonal sensitivity, depression, anxiety, hostility, phobic anxiety, paranoid ideation and psychoticism (11, 12). The current epidemiological data on the prevalence of AAB in China were scarce and insufficient to provide preliminary estimates in the population. Therefore, given the lack of reliable epidemiological information in the current medical literature, the aim of this study was to estimate the prevalence of AAB in China, and to assess the impact of AAB on individuals with AAB, as well as to investigate the attitudes of individuals without AAB toward those with AAB.

## 2. Methods

### 2.1. Survey description

From September 2021 to December 2021, students from several universities/colleges in China were screened with a series of questions to define the AAB population through the Questionnaire Star survey. Questionnaire Star is a free professional online questionnaire survey and evaluation platform that can provide powerful and user-friendly online questionnaire surveys and survey results analysis. The questionnaire sheets were anonymous. The studies involving human participants were conducted in accordance with the Declaration of Helsinki, and were reviewed and approved by the Ethical Review Committee of Taihe Hospital of Hubei University of Medicine (Shiyan, China). The patients/participants provided their written informed consent to participate in this study.

The questions raised in the survey were designed to identify individuals with AAB, regardless of whether they had been diagnosed by a physician, and to define the prevalence and impact of AAB in the sample population. Information about current age, age of onset, gender, ethnicity, family history, and treatment history were also determined. The ascertainment of AAB was based on whether the participant has experienced an unpleasant axillary apocrine odor. Based on the number of higher education students in China in 2021, the number of higher education students with AAB was calculated.

### 2.2. The Chinese version symptom checklist 90

The Chinese version of SCL-90 has good internal consistency and reliability and is widely used in China (12). It also contains nine symptom dimensions with 90 items. Scores for each item range from 1 to 5, indicating no, mild, moderate, little severe, to severe symptoms. Each item is rated based on how distressed the individual was over the past week.

### 2.3. Statistical analysis

SPSS (Statistical Product and Service Solutions) version 23.0 was used to analyze the data. Descriptive statistics frequency and proportion were used to summarize sample characteristics. The *t*-test or *t*’-test was used to analyze the difference in SCL-90 scores between individuals with or without AAB. The *p ≤* 0.05 was considered statistically significant.

## 3. Results

### 3.1. Prevalence and demographic characteristics of AAB in China

A total of 2,625 questionnaires were collected, of which 2,571 were valid questionnaires, with an effective rate of 97.94%. The average age of the students surveyed was 20.46 ± 3.12 years (The age distribution of surveyed students was summarized in Table 1). The surveyed students were mainly from Hubei Medical University (2,149 cases, 83.59%), Hubei University of Arts and Sciences (216 cases, 8.40%) and Ankang University (178 cases, 6.92%). The overall prevalence of AAB among the students surveyed was 7.5% (194/2571) (Table 2). By gender, the prevalence of AAB was 7.0% (110/1566) for female and 8.4% (84/1005) for male. By ethnicity, the prevalence of AAB was 7.35% (175/2382) for Han Ethnicity, 8.7% (6/69) for Tujia Ethnicity, 13.79% (4/29) for Zhuang Ethnicity, 6.67% (1/15) for Miao Ethnicity, 7.69% (1/13) for Hui Ethnicity, and 14.29% (2/14) for Yi Ethnicity (Table 2). The projected number of Chinese higher education students with AAB is about 3 million (more than 40 million college students in China in 2021).

**Table 1:** Age distribution of the surveyed students.

Ages	17y	18y	19y	20y	21y	22y	23y	24y	26y	27y	28y	>28y
Number of students	26	351	672	647	397	157	116	96	35	28	27	19

**Table 2:** The overall prevalence of AAB and the prevalence of AAB in each ethnicity.

Ethnicity	Number of students	Number of students with AAB	Prevalence of AAB
Han	175	2,382	7.35%
Tujia	6	69	8.7%
Zhuang	4	29	13.79%
Miao	1	15	6.67%
Yi	2	14	14.29%
Hui	1	13	7.69%
others	5	49	10.2%
Total	194	2,571	7.5%

The onset age of AAB was as follows, 12.37% (24/194) was under 10 years old, 53.09% (103/194) was between 11 and 15 years old, 26.82% (52/194) was between 15 and 20 years old, 7.22% (14/194) was between 21 and 25 years old, and only 1 case was over 25 years old (Table 3). AAB mainly begins in early adolescence. Among the individuals with AAB, 68.04% (132/194) had a positive family history and 60.30% (117/194) had wet earwax. Sixty-one cases (31.44%) of colored axillary sweat were reported, of which 46 were yellow, 13 were green, 1 was blue, and 1 was brown. Sixty-three cases (32.47%) had received treatment for AAB before the survey, including 24 cases of surgical treatment, 14 cases of drug treatment, 13 cases of laser treatment, and 12 cases of other treatments. As for the treatment response, 8 cases had good response, 20 cases had moderate response, and 10 cases had poor response.

**Table 3:** Age distribution of AAB onset.

Ages	<10y	11–15y	15–20y	20–25y	>25y
Number of students	24	103	52	14	1

### 3.2. AAB negatively affects the sufferers’ daily lives and mental health

The survey from 194 individuals with AAB showed that due to AAB, 37.09% felt depressed and anxious, and 24.88% felt lonely (Table 4). Approximately two-thirds (63.38%) of individuals with AAB worried others knew they had axillary odor, 37.09% did not want to have close contact with others, and 30.05% were unwilling to participate in group activities (Table 4). In addition, 29.58% reported that the disease interfered with daily learning, and 27.23% had no confidence in dating (Table 4). Only 43.19% of those with AAB can take the initiative to talk about AAB with others (Table 4). More than half of the individuals with AAB (53.52%) were eager to treat AAB (Table 4).

**Table 4:** Proportion of individuals with AAB affected by AAB.

Items	Yes (%)	No (%)
Family history	68.04	31.96
Wet earwax	60.30	39.70
Actively talk about AAB	43.19	56.81
Worry that others will know	63.38	36.62
Feel lonely	24.88	69.48
Feel depressed and anxious	37.09	62.91
Unwilling to have close contact with others	37.09	62.91
Unwilling to participate in group activities	30.05	69.95
Affect learning	29.58	70.42
No confidence in dating	27.23	72.77
Eager to treat AAB	53.52	46.48

### 3.3. Attitudes of individuals without AAB toward individuals with AAB

The survey from individuals without AAB showed that 43.45% were willing to keep close contact with individuals with AAB, but only 27.54% were willing to date with individuals with AAB (Table 5). Only 16.90% of individuals without AAB would actively talk about AAB with those who had, but 81.57% reported that they would actively recommend the treatment for AAB to individuals with AAB, and 88.09% reported that they would support individuals with AAB to treat AAB (Table 5).

**Table 5:** The attitude of individuals without AAB to individuals with AAB.

Items	Yes (%)	No (%)
Classmates with AAB	46.52	53.48
Actively talk to individuals with AAB about AAB	16.90	83.10
Willing to be in close contact with individuals with AAB	43.45	56.55
Dating with individuals with AAB	27.54	72.46
Actively recommend treatment to individuals with AAB	81.57	18.43
Support individuals with AAB to receive treatment	88.09	11.91

### 3.4. AAB had negative effects on nine psychological symptoms

Table 6 showed the comparison scores of SCL-90 between individuals with and without AAB (Table 6). Both the *t*-test and *t*’-test showed that the scores of the nine psychological symptom dimension subscales in individuals with AAB were significantly higher than those of individuals without AAB (*p* < 0.001), indicating that AAB had negative effects on the somatization, obsessive compulsive symptoms, interpersonal sensitivity, depression, anxiety, hostility, phobic anxiety, paranoid ideation, and psychoticism.

**Table 6:** Comparison of the SCL-90 primary symptom dimension subscale scores between individuals with and without AAB.

Dimensions	AAB group (M ± SD) *N* = 194	Non-AAB group (M ± SD) *N* = 2,377	*p-*value *t*-test
Somatization	1.73 ± 0.92	1.41 ± 0.59	<0.001
Obsessive compulsive symptoms	2.11 ± 0.90	1.80 ± 0.74	<0.001
Interpersonal sensitivity	1.94 ± 0.89	1.62 ± 0.71	<0.001
Depression	1.87 ± 0.86	1.57 ± 0.69	<0.001
Anxiety	1.77 ± 0.86	1.48 ± 0.65	<0.001
Hostility	1.72 ± 0.78	1.46 ± 0.64	<0.001
Phobic anxiety	1.74 ± 0.86	1.43 ± 0.63	<0.001
Paranoid ideation	1.72 ± 0.85	1.43 ± 0.63	<0.001
Psychoticism	1.77 ± 0.90	1.44 ± 0.63	<0.001

## 4. Discussion

AAB is a functional disease, but the malodor can lead to serious social and psychological problems to the sufferers (2). The diagnostic criteria for AAB have not been established (13). A phenotype-based diagnosis according to a positive family history and presence of wet earwax, and a genotyping-based diagnosis according to alleles 538 GG or GA, both are proposed (14–16). However, not all individuals with a positive family history, wet earwax, or alleles 538 GG or GA suffer from AAB, and vice versa (17). In addition to genetics, AAB is affected by several other factors, such as age, gender and diet (18, 19). Body odor tends to increase with age (20). Body odor develops in children at the onset of puberty, and the unpleasant greasy and grassy smell of 2-nonenal was detected only in subjects aged 40 years or older (20). Dietary intake may alter the type and amount of axillary fatty acid secretion and lead to changes in odor severity (18). The gender difference may be due to men producing more sweat than women, which enhances the diffusion of odor (19). Human body odor is affected by a variety of odor components. These factors do not work in isolation but interact with each other (18, 19). Generally, genetics affects the occurrence and severity of AAB, and other factors only affect the severity of AAB (18, 19). Chinese people have very faint body odor and are sensitive to odor, so it is not difficult to judge if a person suffers from AAB. In the study, we survey the epidemiological data on the prevalence of AAB in China, assess the impact of AAB on individuals with AAB, and investigate the attitudes of AAB-free individuals toward AAB individuals.

We first investigate the prevalence of AAB in China and preliminarily estimated the number of Chinese higher education students suffering from AAB. We choose higher education students for the survey mainly based on the following three reasons. One is that the occurrence of AAB depends on the function of the apocrine sweat glands, and the apocrine sweat glands do not work until puberty. Puberty usually occurs between the ages of 10–14 years in girls and 12–16 years in boys, and lasts about 2–3 years. In China, most people start higher education at the age of 18 years. Body odor is more pronounced in this age group due to fully developed apocrine glands and increased physical activity. Moreover, people in this age group have just entered adulthood, have further requirements for interpersonal relationships, and pay more attention to their body odor. The other is that the gross enrollment rate of higher education in China reached 54.4% in 2020,^1^ so the higher education population can represent the main demographic characteristics of the same age group. Another is that the survey is conducted online, diagnosing AAB mainly based on whether an individual has experienced unpleasant pungent underarm odors. Compared with junior and senior middle school students, higher education students have matured and reliable cognitive and judgment ability, so they can more accurately judge whether they have underarm pungent odor. The overall prevalence of AAB among the survey students was 7.5%. By September 2021, more than 40 million students were pursuing higher education in mainland China (see text footnote 1).^2^ The projected number of Chinese high education students with AAB is about 3 million.

Many studies have shown that AAB is inherited in an autosomal dominant manner, with no gender preference (8, 17). In our survey, the prevalence of AAB was 7.0% in female and 8.4% in male, with a higher prevalence in male than in female. The difference in the prevalence of AAB between male and female may be that male tends to have stronger body odor than female and are therefore easier to identify (21). A study by Nakano et al. showed that AAB was strongly associated with the wet earwax genotype by comparing the frequency of the rs17822931 genotype in Japanese population with AAB and the general population (17). In their study, approximately 98.7% (78/79) of individuals with AAB had 538 GG or GA, while only 35.4% (57/161) of the general population had this genotype (17). The 538 AA is common (80–95%) in East-Asians, such as Korean, Chinese, and Japanese, and rare (0–3%) in Europeans or Africans (9, 17, 22, 23). Our survey data on individuals with AAB shows that 68.04% had a positive family history and 60.30% had wet earwax. A study by Zhu et al. showed that 72.73% (24/33) of individuals with a clinical diagnosis of AAB had a positive family history, which were similar to ours (18).

In addition to heredity, AAB is also race/ethnicity (24, 25). Many studies had shown that the prevalence of AAB tended to be lower in East Asian descent than in African and European descent, and the prevalence was higher in dark-skinned groups than in other groups (2, 3). In the study, when further subdivided by ethnicity, the prevalence of AAB in Ethnic Han was lower than that of Ethnic Tujia and Ethnic Zhuang. Although the prevalence of AAB in Ethnic Miao, Hui and Yi had also been counted, but the base value was too small to reflect the actual situation. Previous study has shown that Han Ethnic majority in China have a gene that reduces the likelihood of developing AAB (23).

The survey in the study showed that most of the onset age was between 11 and 20 years old (79.90%, 155/194), which was in line with the physiological process of human apocrine development and coincides with previous reports (2, 26). Before the survey, approximately one-third individuals with AAB had received surgery, drugs, lasers and other treatments, indicating that individuals with AAB often seek treatments. Studies among different ethnic groups showed that Asians usually seek treatment for AAB, possibly because only a minority of Asians suffer from AAB and most Asians consider the odor offensive (2). As for the treatment response in the survey, 73.68% (28/38) had moderate to good response, while 26.32% (10/38) had poor response. Although there are many treatments for AAB, the ideal treatment remains to be explored (2, 27, 28).

Second, we assess the impact of AAB on individuals with AAB, and how individuals without AAB think of individuals with AAB. The results showed that AAB had profound detrimental effects on an individual’s daily life, learning and social communication, as well as mental and psychological health. Individuals with AAB often felt depression, anxiety, loneliness, social alienation, and sometimes become social dropouts. About a quarter of individuals with AAB were not confident about dating, and correspondingly, only a quarter of individuals without AAB were willing to date those with AAB. Nonetheless, about half of the individuals without AAB did not mind being in close contact with those with AAB. The reason may be that Asians tend to have milder body odors, and the unpleasant odor of AAB is often suffocating, so individuals with AAB are often not accepted by families without AAB (2). Influenced by traditional Chinese culture, Chinese people are relatively introverted, so whether the individuals with or without AAB, they will not take the initiative to talk about AAB with each other. In contrast, both individuals with and without AAB expect AAB can be treated.

Third, we survey and compare the mental health status of individuals with or without AAB. The SCL-90 is a popular tool for assessing mental health and has proven useful (29). Many literatures had mentioned the mental and psychological damages of AAB to the sufferers, but few studies were conducted on it in English-language literatures (2, 30). In the study, the scores of the nine psychological symptom dimension subscales in individuals with AAB were significantly higher than those of individuals without AAB. The data indicated that AAB had detrimental effects on individuals’ mental health.

Taken together, the prevalence estimates of AAB provided by this survey shows that although only a small proportion of the Chinese population suffers from AAB, the number is large. For individuals with AAB, the unpleasant odor often interferes with their daily activities and leads to emotional, social, and psychological impairments. Most individuals with AAB have been treated or want to be treated. While a range of treatment options are available, more effective treatments with fewer complication are needed. China and the West or East-Asia and the West have different perception, recognition and treatment preferences for AAB.

## Data availability statement

The original contributions presented in the study are included in the article/supplementary material, further inquiries can be directed to the corresponding authors.

## Ethics statement

The studies involving human participants were conducted in accordance with the Declaration of Helsinki, and were reviewed and approved by the Ethical Review Committee of Taihe Hospital of Hubei University of Medicine (Shiyan, China). The patients/participants provided their written informed consent to participate in this study.

## Author contributions

LZ, HL, and CZ conceived and designed the surveys. LZ, JC, CW, JZ, and HL conducted the surveys. LZ and HL analyzed the data and wrote the manuscript. All authors contributed to the article and approved the submitted version.

## Footnotes

^1^www.moe.gov.cn

^2^www.chinadaily.com.cn
